# Combination of beneficial bacteria improves blueberry production and soil quality

**DOI:** 10.1002/fsn3.1772

**Published:** 2020-09-23

**Authors:** Yi‐Yang Yu, Jing‐Da Xu, Tao‐Xiang Huang, Jian Zhong, Hong Yu, Jing‐Ping Qiu, Jian‐Hua Guo

**Affiliations:** ^1^ Department of Plant Pathology College of Plant Protection Key Laboratory of Monitoring and Management of Crop Diseases and Pest Insects Ministry of Agriculture Nanjing Agricultural University Nanjing China; ^2^ Institute of Botany Jiangsu Province and Chinese Academy of Science Nanjing Botanical Garden Mem. Sun Yat‐Sen Nanjing China

**Keywords:** beneficial bacteria consortium, blueberry, organic system, soil quality, yield

## Abstract

Blueberry is an important agricultural crop with high nutritional, health, and economic value. Despite the well‐studied blueberry cultivation methods and soil requirements, little is known about how beneficial bacteria function in organic blueberry cultivation systems and their effects on acidic soils. In this study, a single bacteria *Bacillus amyloliquefaciens* JC65 and three biocontrol bacteria consortiums containing JC65 were applied to organic system. The effect of bacteria to blueberry growth, yield, fruit quality, and soil quality was investigated. A consortium of three mixed *Bacillus* (*B. amyloliquefaciens* JC65, *B. licheniforims* HS10 and *B. subtilis* 7ze3) showed the highest growth improvement efficiency. The bacterial inoculation increased blueberry leaf chlorophyll content, net photosynthetic rate by 21.50%, 13.21% at 30 days, and increased average plant height by 2.72% at 69 days. Compared with the control, the inoculated plants showed an increased yield of 14.56%. Interestingly, blueberry fruit quality was also improved with supplement of the bacterial consortium. Fruit anthocyanin, soluble sugar, vitamin C, soluble solids, and soluble protein content were increased by 5.99%, 4.21%, 17.31%, 2.41%, and 21.65%, respectively. Besides, beneficial bacterial consortium also enables sustainable agriculture by improving soil ammonium nitrogen and organic matter by 3.77% and 2.96% after blueberry planting. In conclusion, the combination of beneficial bacteria showed a synergistic activity in organic system to promote the blueberry yield, fruit quality, and soil nutrient preservation.

## Graphical abstract

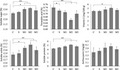

## 1 INTRODUCTION

Blueberry is attracting keen interest all over the world for its high nutritional value, rich flavors, and health properties. As a result of consumer's well receiving of healthy food, world blueberry production has increased rapidly over the recent decades. With the development of the economy and the awareness of the people's health care, China's blueberry production has rapidly prospered from a small industry to a leader in the Asia‐Pacific region in just a few years. In 2016, China's blueberry planting acreage accounts for more than 20% of the world's total, and produce around 20 thousand tons of fresh blueberry (Brazelton & Young, 2014). Even so, the growth of blueberry production still lags behind the consumers’ demand (Villata, 2012). Therefore, optimizing blueberry production conditions and increasing blueberry yield are of great significance to society and economy.

Most consumers have a positive attitude toward organic foods, and the market prospects of healthy organic foods are generously optimistic (Kihlberg & Risvik, 2007). Specifically, consumers who are interested in purchasing blueberry as a health‐friendly fruit are often willing to pay for additive value of organic blueberry as a “reduced‐risk” product (Drummond, Smagula, Annis, & Yarborough, 2009). Besides benefits on food safety, organic farming system enables formation of a benign cycle based on a healthy ecological environment by improving biodiversity, soil environment, and protecting the ecological environment. Broad‐spectrum insecticides, for instance, are effective in controlling agricultural pests. However, they also threaten the living and multiply of natural enemies and seriously endanger the agricultural ecological environment. On the contrary, products approved for use in organic agriculture are compatible with beneficial insects, which in turn support natural biological pest management (Roubos, Rodriguez‐Saona, Holdcraft, Mason, & Isaacs, 2014). Moreover, compared with other crops, blueberry has unique advantages in embracing organic cultivation because of its relatively high vigor and resistance to pests due to a short domestication history of <100 years (Sciarappa et al., 2008).

Although organic blueberry production has become an important part of the blueberry industry worldwide, in most areas, organic blueberry production still faces many challenges, such as increased production costs or inputs (especially in fertilization and weed management in an organic way), limited choice of disease or pest control methods, and low production of organic blueberries (Pretty, 1997). Beneficial microbe, often known as plant growth‐promoting rhizobacteria (PGPR), is considered to be one of the solutions to those problems. PGPR participate in multiple physiological processes of plants including establishment of plant morphology, plant growth, cycling of nutrient, and disease defense (Wu, Cao, Li, Cheung, & Wong, 2005). Some of the PGPR have been reported to induce plant systemic resistance against both pathogen and insect by stimulating SA and JA/ET pathway (Niu et al., 2011). In another study, application of two *Bacillus* strains, OSU‐142 and M3, has shown positive effect on raspberry growth and yield (Orhan, Esitken, Ercisli, Turan, & Sahin, 2006). Besides beneficial effect on plant, PGPR also have the potential to alter soil properties in the long term. Secretions of *B. cereus* AR156 are able to influence plant root secrets (Zhou et al., 2016). The change of root secrets has effects on plant growth; moreover, it provides more organic matter, such as lactic acid and caproic acid, for the soil (Wang et al., 2019). It has also been shown that PGPR are able to promote soil water retention (Zheng et al., 2018). Nevertheless, information about the effect of PGPR on blueberry growth, yield, and soil nutrients in organic planting system is still scarce.

In this study, we evaluated the effect of single and complex beneficial microbe on blueberry growth, yield, fruit quality as well as soil content after one season of planting in an organic cultivation system by analyzing the major corresponding parameters.

## 2 MATERIALS AND METHODS

### 2.1 Preparation of bacteria consortium

*Bacillus amyloliquefaciens* JC65, *B. subtilis* 7ze3, *B. subtilis* JC03, and *B. licheniforims* HS10 were grown at 28°C in LB medium for 24 hr. The four bacterial cultures were then adjusted to ~5 × 10^7^ cfu/ml with water. The diluted bacteria culture was mixed by the ratio of 1:1:1 in M1 (JC65 + 7ze3 + JC03), M2 (JC65 + 7ze3 + HS10), and M3 (JC65 + JC03 + HS10).

### 2.2 Field experiment

The field trial began in December 2016 and last for 2 years. The experiment site is located at Lanmei agricultural ecological park, Kuizhang village, Nanjing city (E 118° 22′ 51.67′′, N 31° 56′ 12.14′′). The blueberry variety used in this trial is “Lanmei No.1”, which was selected by Zhejiang Lanmei Agricultural Co., Ltd. The blueberry seedlings were transplanted in 2016.

Five treatments were carried out in this study, including a control treatment C, for an organic system with no addition of beneficial microbe; treatment S, for an organic system supplemented with single beneficial bacteria *B. amyloliquefaciens* JC65; treatment M1, M2, and M3, for organic systems supplemented with mixed beneficial bacteria of JC65 + 7ze3 + JC03, JC65 + 7ze3 + HS10, and JC65 + JC03 + HS10, respectively. We used JC65 as the core strain in the formula because, firstly, JC65 had previously been reported to have significant promotion effect on the watermelon's resistance to *Acidovorax avenae* subsp. *citrulli*, as well as on plant growth (Jiang et al., 2015). The purpose of this experiment design was to screen for more effective consortiums in promoting organic blueberry growth by using the beneficial microorganism JC65 as the core strain with two other alternate *Bacillus* environmental isolates. The feasibility of the three‐strain mixtures was analyzed by testing the difference between the effects of single bacteria and different consortiums on blueberry production. Moreover, we also wanted to summarize the preliminary rules of bacteria mixture to guide the development of other bacteria consortiums by comparing the effects of different three‐strain consortiums.

In 2016, well‐ventilate field with loose soil, high organic content, and no water accumulation was chosen for experiment. One tone per hectare of sulfur and organic compost made from peat, wood chips, rotten, and pine crust were applied into the selected field. Soil was covered with black film to maintain soil temperature and humidity. Dry straw was placed in the furrows as cover crop to help control the grass. Blueberries were planted on mounds with a space of 1.5 m, and plant spacing of each blueberry was 1.2 m. Approximately 3,750 seedlings were planted per hectare. One mound of the field with 30 seedlings was set as one treatment. Each treatment was repeated 3 times on the mounds randomly distributed in the whole field. In February 2017 and February 2018, organic fertilizer, which was mainly composed of sheep manure, was applied into the field with an amount of 7,500 kg/ha. Drip irrigation was employed in each system to control soil moisture and prevent the disease‐leading excessive humidity. Manual weeding was equally carried out in all tested organic systems. Sweet and sour liquid (sugar: vinegar: wine: juice: clear water = 1.5:1:1:1:5) was applied near the orchard to trap adult flies and chafers during mature and harvesting period (from late May to July 2018). Diluted 0.6% matrine water solution was sprayed 600 ml/ha to control pests such as flies. Blueberry fruit was picked by hand. In order to maintain strong shoots and optimize fruit quality rather than maximizing yield, trees were pruned during winter.

### 2.3 Determination of growth index

In each treatment, 24 plants from the three repeat mounds were randomly selected. Plant height was measured with a tape measure. The leaf chlorophyll content was determined by chlorophyll meter (TYS‐A) from Zhejiang Top Yunnong Technology Co., Ltd. The net photosynthetic rate of the leaves of 9 randomly selected blueberry trees for each treatment was determined with LI‐COR Li‐6400 photosynthetic apparatus.

### 2.4 Soil sampling and determination

Soil samples were taken before treatment (December 2016) and after harvest (August 2018). Soil samples were collected according to the five‐point sampling method at the same sampling depth of about 10–15 cm below the ground. Plant roots and small rocks were removed and the soil was air‐dried. After grinding, the ammonium nitrogen (AN), available phosphorus (AP), and available potassium (AK) were determined with a soil tester (TPY‐6A type), and the pH value of the soil was measured with a pH meter (Ray‐Magnetic PHS‐3C type). Soil EC values were determined using a conductivity meter (Bante 950). Soil organic matter (OM) percentage was determined with oil bath heated potassium dichromate oxidation‐reduction titration (Schollenberger, 1931).

### 2.5 Fruit quality determination

For each treatment, 15 blueberry fruits with similar size and hardness were selected and squeezed. The juice was mixed thoroughly and sampled 3 times to determine soluble sugar with a saccharimeter LB32T and soluble solids with a refractometer ATAGO PAL‐1. One gram of fruit flesh was sampled and ground from five fruits with similar size and hardness in each treatment. Vitamin C was determined with ultraviolet spectrophotometry (Santos, Lima, Março, & Valderrama, 2016). The Coomassie Brilliant Blue G‐250 staining method was used to determine the soluble protein content (Jones, Hare, & Compton, 1989). The titratable acid content was determined by phenolphthalein titration (Alamo, Maquieira, Puchades, & Sagrado, 1993). Fourteen fruits with similar maturity were lyophilized and ground in liquid nitrogen. Two grams of ground material was collected to determine total anthocyanin content (Solomakhin & Blanke, 2010). Assays were repeated three times.

### 2.6 Statistical analysis

Data were analyzed using Statistical Analysis Software (SAS Institute). One‐way analysis of variance (ANOVA) was used. Means were separated using the least‐significant difference test (LSD).

## 3 RESULTS

### 3.1 PGPR effects on blueberry growth and yield

Thirty days after treatment, the growth index of blueberry, chlorophyll content, and net photosynthetic rate of blueberry leaves were measured. Compared with the organic cultivation control C, the composite bacteria treatment (M1, M2, M3) increased the chlorophyll content of the blueberry leaves by 15.33%, 21.50%, and 9.47%, respectively. There was no significant difference on chlorophyll content between the single bacterial treatment S and the control C. Moreover, M1, M2, and M3 treatments increased the average net photosynthetic rate of blueberry leaves by 11.79%, 13.21%, and 9.65%, respectively, compared with the control C. However, due to a substantial in‐treatment variation of leaves, no significant difference on net photosynthetic rate was found among the treatments (Table 1). Sixty‐nine days after treatment, blueberry growth index and leaves chlorophyll content were also recorded. Compared with the control, the composite bacteria treatment (M1, M2, M3) increased the chlorophyll content of the blueberry leaves by 1.47%, 3.03%, and 2.49%, respectively. In addition, M1, M2, and M3 increased the average height of blueberry than control C by 2.40%, 2.72%, and 2.62%, respectively, but without significant difference between treatments (Table 1).

**Table 1:** Growth indexes of blueberries at different time periods after treatment

Treatment	0 day aftertreatment	30 days after treatment			69 days after treatment	
	Plant height (cm)	Plant height (cm)	Chlorophyll (SPAD)	Net photosynthetic rate (μmol CO_2_ m^−2^ s^−1^)	Plant height (cm)	Chlorophyll (SPAD)
C	92.00 ± 0.75A	93.25 ± 0.96A	34.32 ± 0.33D	6.300 ± 0.466A	93.83 ± 0.48A	57.05 ± 0.16BC
S	92.00 ± 0.71A	93.75 ± 1.15A	35.20 ± 0.51D	6.225 ± 0.326A	94.58 ± 0.77A	57.02 ± 0.70C
M1	92.00 ± 0.81A	94.13 ± 0.52A	39.58 ± 0.62B	7.043 ± 0.644A	96.08 ± 1.16A	57.89 ± 0.32ABC
M2	92.17 ± 0.95A	93.79 ± 0.82A	41.70 ± 0.27A	7.132 ± 0.558A	96.38 ± 1.68A	58.78 ± 0.27A
M3	92.00 ± 0.73A	93.88 ± 0.65A	37.57 ± 0.57C	6.908 ± 0.523A	96.29 ± 1.17A	58.47 ± 0.20AB

Note

Data are showed as mean ± standard deviation. The values with different uppercases are significantly different among different treatments at *p* < .01. C: blank control; S: JC65; M1: JC65 + 7ze3 + JC03; M2: JC65 + HS10 + 7ze3; M3: JC65 + JC03 + HS10.

The total yield of blueberry was also calculated after harvest. M1, M2, and M3 improved blueberry yield by 3.45%, 14.56%, and 6.78% compared with the control C, respectively. The yield of blueberry treated with M2 is significantly higher than that of other treatments. However, single bacterial treatment S showed no significant yield improvement than the control. No significant difference on single fruit weight was shown among all treatments (Figure 2).

### 3.2 PGPR effects on blueberry fruit quality

The quality of blueberry fruit, including soluble sugar, titratable acid, vitamin C, soluble solid, soluble protein, and anthocyanin content, was also determined. Results showed that compared with the control C, the compound bacteria treatment M1, M2, and M3 significantly improve the fruit quality of blueberries. Among all, the M2 treatment showed the best effect on fruit quality with an increase on soluble sugar, vitamin C, soluble solids, and soluble protein content by 4.21%, 17.31%, 2.41%, and 21.65% (Figure 2), respectively. Moreover, the titratable acid was reduced by 10.81% in M2 treatment (Figure 2). The average anthocyanin content of M2 treatment was also 5.99% higher than control. The single‐strain treatment S also increased the quality of blueberry fruit by 1.03% on average soluble sugar content and 5.88% on average soluble protein (Figure 2). However, this increase was not statistically significant.

### 3.3 PGPR effects on soil quality

We collected soil samples before and after harvest and determined the basic physical and chemical properties of the soil. The soil properties changed after one season of planting in organic system, which was used as control in this study. The content of ammonium nitrogen in the soil decreased 20.64% in the control, while an increase of 3.77% was detected in M2 treatment (Figure 3). Soil organic matter decreased 6.17% in the control organic system by the harvest time; on the contrary, organic matter content in soil increased significantly in M1, M2, and M3 treatment after planting by 2.07%, 2.79%, and 2.04%, respectively (Figure 3). For available potassium and phosphorus content, although decreased in all the treatments, the decrease rate in M2 treatment was significantly lower than other treatments. In the single bacterial treatment S, the decline rate of soil organic matter content was significantly lower than that of control treatment; however, no other significant difference on soil physical and chemical properties in this treatment was detected compared with the control C treatment (Table 2). Soil EC value decreased at harvest in C treatment, while in composite bacteria treatment, especially in M2 and M3, the drop was more significant.

**Table 2:** Soil indexes before and after treatment

Treatment		pH	EC (µS cm^−1^)	AN (‰)	AK (‰)	AP (‰)	OM (%)
C	Before treatment	6.38 ± 0.01a	355.00 ± 14.11a	26.61 ± 0.59ab	140.40 ± 1.40ab	39.97 ± 0.19a	2.14 ± 0.02a
	After treatment	5.58 ± 0.01b	324.00 ± 14.11b	18.27 ± 0.37e	129.73 ± 2.91d	28.97 ± 0.82d	2.01 ± 0.02c
S	Before treatment	6.38 ± 0.01a	373.33 ± 29.74a	25.84 ± 0.53b	141.50 ± 2.31a	40.03 ± 0.09a	1.99 ± 0.07c
	After treatment	5.58 ± 0.02b	315.67 ± 10.69b	20.50 ± 0.40d	129.50 ± 6.84d	34.97 ± 0.19c	1.99 ± 0.02c
M1	Before treatment	6.38 ± 0.02a	358.00 ± 16.70a	26.27 ± 0.87b	141.23 ± 2.71a	39.95 ± 0.43a	2.00 ± 0.06c
	After treatment	5.58 ± 0.02b	283.67 ± 8.50c	24.61 ± 0.79c	135.57 ± 3.39bc	35.12 ± 0.14c	2.05 ± 0.02bc
M2	Before treatment	6.38 ± 0.01a	365.67 ± 19.14a	26.55 ± 0.52ab	140.23 ± 0.96ab	40.06 ± 0.16a	2.03 ± 0.05bc
	After treatment	5.58 ± 0.01b	263.67 ± 10.60c	27.55 ± 0.96a	140.23 ± 2.80ab	37.29 ± 1.07b	2.09 ± 0.02ab
M3	Before treatment	6.37 ± 0.01a	369.00 ± 27.22a	26.54 ± 0.56ab	139.23 ± 1.21ab	39.96 ± 0.45a	2.03 ± 0.04bc
	After treatment	5.57 ± 0.01b	271.33 ± 14.98c	26.54 ± 0.44ab	133.23 ± 3.83cd	35.56 ± 0.77c	2.07 ± 0.01b

Note

Data are showed as mean ± standard deviation. The values with different lowercases are significantly different among different treatments at *p* < .05. C: blank control; S: JC65; M1: JC65 + 7ze3 + JC03; M2: JC65 + HS10 + 7ze3; M3: JC65 + JC03 + HS10.

Abbreviations: AK, available potassium; AN, ammonium nitrogen; AP, available phosphorus; OM, organic matter.

## 4 DISCUSSION

**FIGURE 1:**
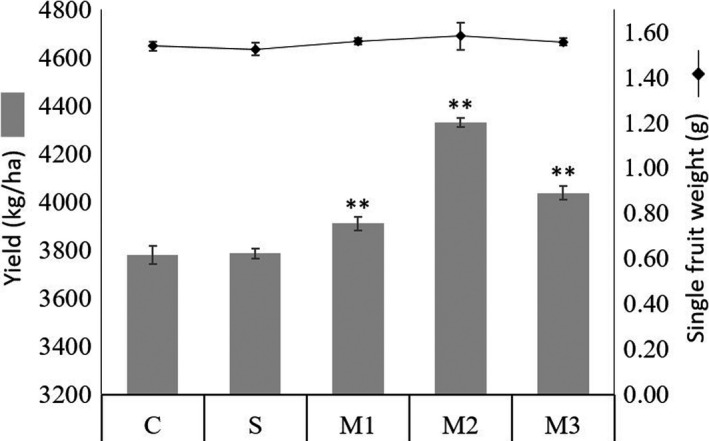
Blueberry yield (gray columns) and single fruit weight (connected scatter plots) under different treatments. C: blank control; S: JC65; M1: JC65 + 7ze3 + JC03; M2: JC65 + HS10 + 7ze3; M3: JC65 + JC03 + HS10. Significant differences are marked as: “**” for *p* < .01

Successful symbioses of plant and microbe, such as leguminous plants and rhizobia, have long been recognized to benefit plant health and growth on various aspects. For highbush blueberry (*Vaccinium corymbosum* L.), ericoid mycorrhizae spontaneously colonize blueberry roots and form mutually beneficial symbiotic relationship (Smith & Read, 2008). Ericoid mycorrhizal promote the plant growth by degrading the soil organic nutrient and delivering it to mycorrhizal plant (Read, Leake, & Langdale, 1989), for instance, producing acid extracellular protease to break down soil proteins into blueberry available N sources (Bajwa & Read, 1985). However, little is known about the effect of PGPRs on blueberry planting. PGPRs have been shown to have beneficial effect on plant by controlling plant disease by either indirectly repressing pathogens or inducing plant systemic resistance, helping to improve soil quality or directly motivating plant growth by activating changes on expression profiles of plant growth‐related genes (Bhattacharyya & Jha, 2012). There have been reports that PGPRs could be applicable agents against plant disease in blueberry production. With a targeted delivery by honeybee, *B. subtilis* effectively control mummy berry disease by suppressing growth of the causal agent *Monilinia vaccinii*‐*corymbosi* (Dedej, Delaplane, & Scherm, 2004; Scherm, Ngugi, Savelle, & Edwards, 2004). In our study, we compared blueberry growth and production in organic agriculture system with or without different bacteria treatments. We found that addition of beneficial bacteria into organic blueberry cultivation system promotes blueberry plant growth and significantly increases blueberry yield, especially in M2 (7ze3 + JC65 + HS10) treatment, in which a 14.56% higher blueberry yield was achieved (Figure 1). Jha and Subramanian (2018) have reported that the accumulation of soluble sugars in plants negatively regulates plant photosynthesis and sugar biosynthesis, while inoculation with beneficial microorganisms increases the accumulation of soluble sugar in plants by enhancing photosynthesis. Similarly, we found that leaf chlorophyll content in M2 treatment was significantly higher than the control treatment (Table 1). Additionally, mean net photosynthetic rate in M2 treatment was also 13.21% higher than the control (Table 1). These results suggest that beneficial bacteria, especially bacterial consortium M2, have the ability to induce photosynthesis and consequently result in higher growth indexes and yield. The improved growth and yield are in consistent with former report that *B. amyloliquefaciens* JC65 (former name 54) was able to promote growth of watermelon (Jiang et al., 2015). However, the growth promotion effect of JC65 in this study is 2.72% which is much lower than the total protein promotion rate of 110% reported on watermelon. This may be due to the limited experimental period compared with growth cycle of perennial woody plant. Alternatively, the acidic soil condition required for satisfactory growth of blueberry (Harmer, 1945) may also influence the growth and the plant growth‐promoting effect of bacteria. In the future, screening for PGPRs that are compatible with acidic soil will be a potential direction to improve the efficacy of PGPRs utilization on blueberry production.

**FIGURE 2:**
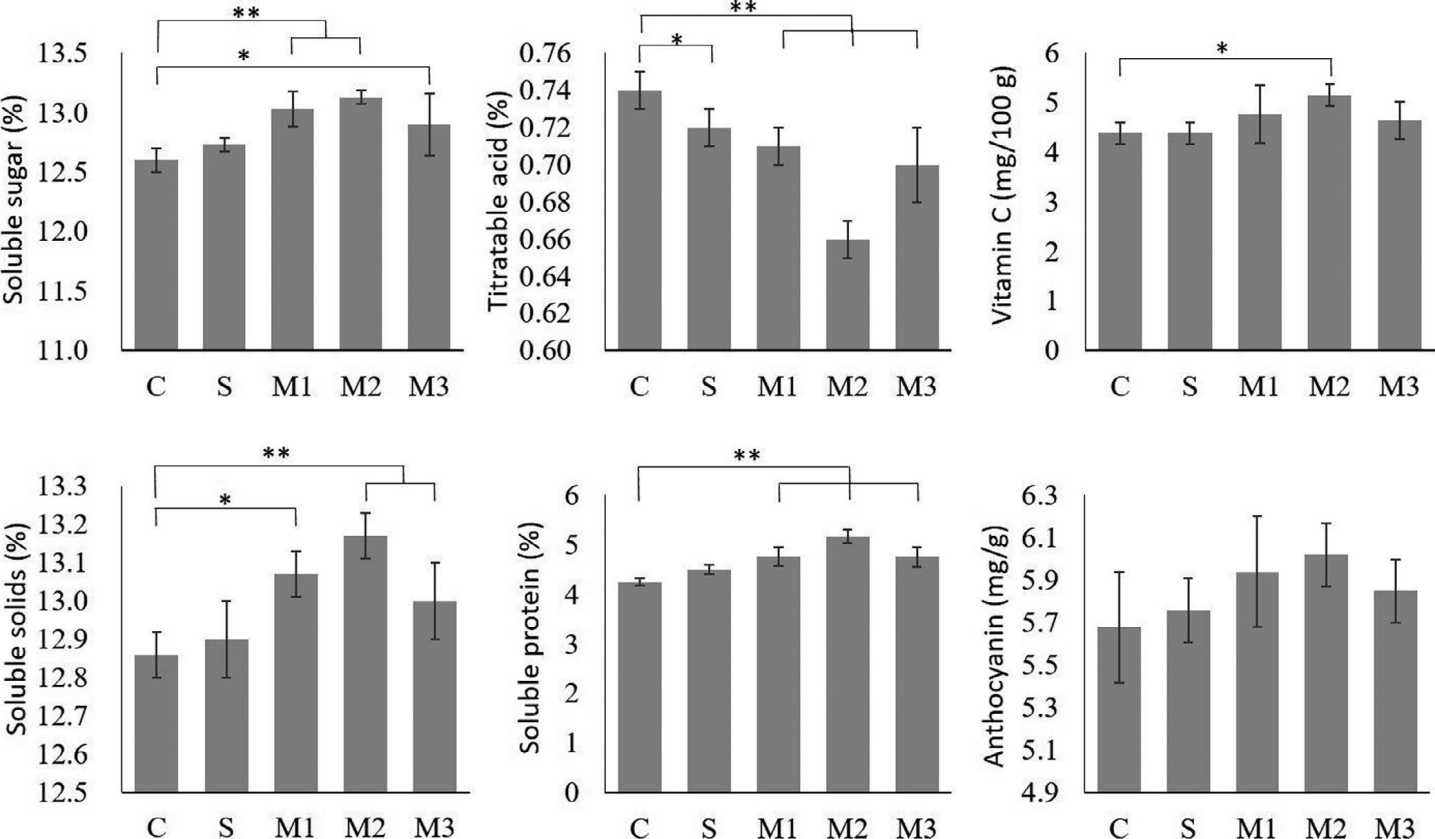
Soluble sugar content (%), titratable acid content (%), vitamin C content (mg/100g), soluble solids content (%), soluble protein content (%), and anthocyanin content (mg/g) of blueberry under different treatments. C: blank control; S: JC65; M1: JC65 + 7ze3 + JC03; M2: JC65 + HS10 + 7ze3; M3: JC65 + JC03 + HS10. Significant differences are marked as: “*” for *p* < .05, “**” for *p* < .01

Blueberry fruit is not only a delicious fruit, but also a health food. Its rich variety of substances, such as polyphenolic compounds, especially anthocyanins, polysaccharides, and triterpenoids, has been proven to have health benefits in many aspects. Anthocyanins, as strong antioxidants and anti‐inflammatory agents for mammalian cells (Bornsek et al., 2012; Krikorian et al., 2010), are shown to be capable of reversing the course of neuronal and behavioral aging (Joseph et al., 1999), inhibiting cancer cell proliferation and invasion (Faria et al., 2010), preventing obesity, other metabolic syndrome disorders (Norberto et al., 2013), etc. Our results show that addition of *Bacillus* promote average anthocyanin content of blueberry fruit with a highest increase of 5.99% in the M2 treatment (Figure 2). Besides the differences among blueberry varieties, the anthocyanin content of blueberry is affected by many factors including light, pH, moisture, fertilization, geographic location, and sampling time during the planting process (Akerström, Forsum, Rumpunen, & Jäderlund, 2009; Akerström, Jaakola, Bång, & Jäderlund, 2010; Rieger, Müller, Guttenberger, & Bucar, 2008). Interestingly, it has been reported that application of plant defense regulator, such as methyl jasmonate, positively affects anthocyanins content in blueberry, however, at a cost of yield loss (Percival & MacKenzie, 2007). Ample research indicates that some bacteria in *Bacillus* sp. are capable to induce plant systemic resistance (Niu et al., 2011) by releasing resistance‐inducing compounds such as extracellular polysaccharides (Jiang, Fan, Xie, & Guo, 2016) and lipopeptides (Ongena et al., 2007). Above reports offer a feasible explanation that the promotion of anthocyanin content achieved in organic planting system supplemented with single or mixed *Bacillus* could be at least partially caused by beneficial bacterial induced plant defense.

Fruit‐related index is also important for quality of blueberry. The additional bacteria in M2 treatment increased the soluble sugar content of blueberry fruit by 4.21%, the content of vitamin C by 17.31%, the content of soluble solids by 2.41%, and the content of soluble protein by 21.65%, while titratable acid in fruit was reduced by 10.81% (Figure 2). These results are consistent with the previously report, in which the application of the microbial consortium improved the fruit quality of pepper (Yu et al., 2019). It has also been reported that the beneficial microorganisms are able to increase the accumulation of soluble sugar in plants by enhancing photosynthesis (Jha & Subramanian, 2018). It is noteworthy that BBP3‐1, a blueberry polysaccharide, is shown to inhibit tumor progression and has the potential to be an immunomodulatory (Sun, Liu, Wu, Feng, & Meng, 2015). Therefore, the improvement of soluble sugar not only promote fruit flavor, but may also promote blueberry health function.

**FIGURE 3:**
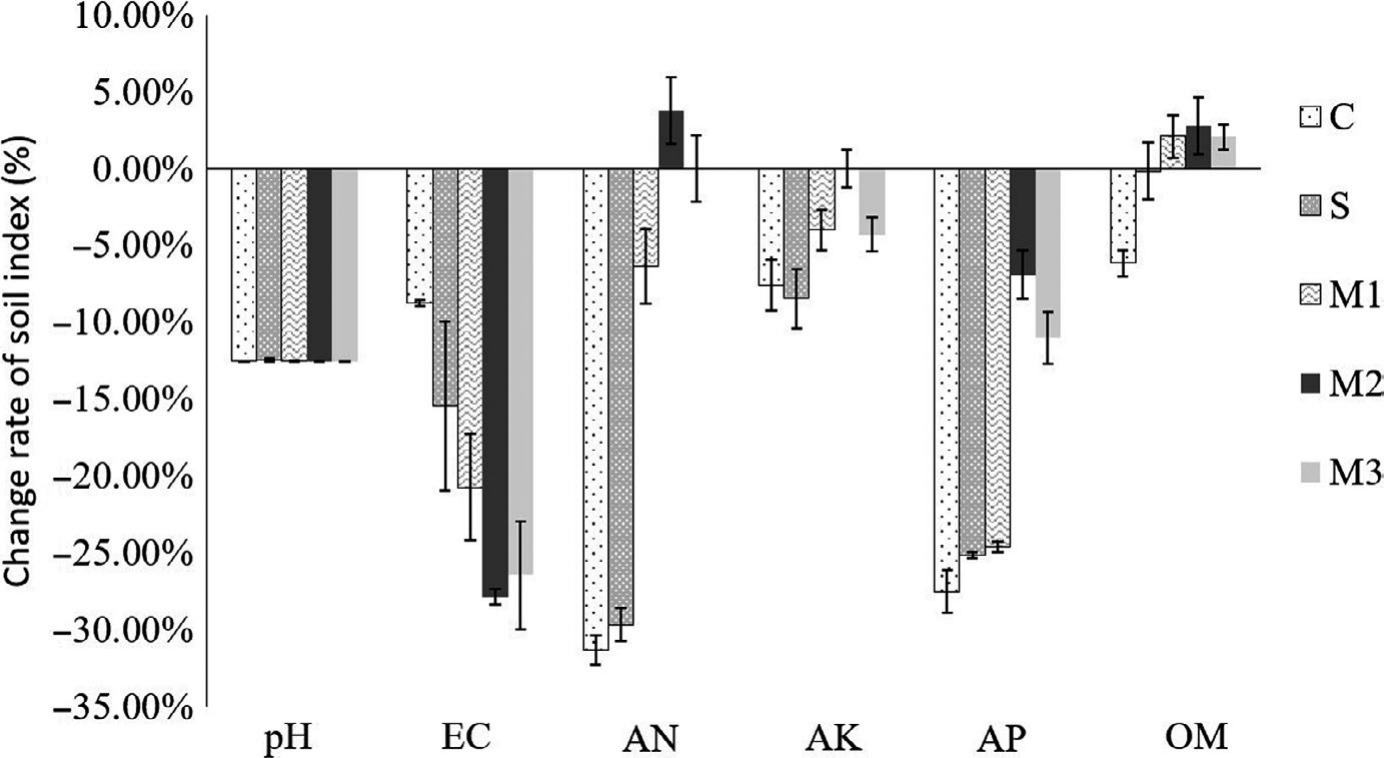
Change rate of soil indexes in different treatments after one season of organic cultivation of blueberry. C: blank control; S: JC65; M1: JC65 + 7ze3 + JC03; M2: JC65 + HS10 + 7ze3; M3: JC65 + JC03 + HS10. AN, ammonium nitrogen; AK, available potassium; AP, available phosphorus; OM, organic matter

Soil quality has a long‐term impact on crop growth. Plant, grown in organic farms, often suffers from limited soil nutrient supply, as addition of chemical fertilizers is restricted in organic systems (Kitchen, McDonald, Shepherd, Lorimer, & Graham, 2003). Understanding on the soil quality dynamics is important to reveal crop nutrient condition and assess sustainability of individual cropping systems in agricultural practices. Nitrogen, phosphorus, and potassium are commonly recognized as the three most important nutrients in plants. NPK fertilization was also found to have positive influence on blueberry yield (Starast, Karp, Vool, Paal, & Albert, 2007). In this study, we compared soil nutrient changes after one season of planting in organic planting system motivated by single bacterium JC65 (S), microbial consortium JC65 + 7ze3 + JC03 (M1), JC65 + 7ze3 + HS10 (M2), and JC65 + JC03 + HS10 (M3) with control organic planting system (C) (Table 2). The reduction in soil nutrient after one season of planting was significantly less in beneficial microbe motivated organic planting system compared with the control organic planting system (Figure 3), indicating the soil nutrient preservation effect of the beneficial microbe. These results are supported by the reported ability of soil‐dwelling *Bacillus* to increase soil available nitrogen, phosphorus, and potassium nutrition. Wang, Liu, and Li (2014) have shown that, in acidic soil condition, *Bacillus* increases the content of available phosphorus by converting insoluble phosphorus into soluble ions. Similarly, as reported by Sheng and He (2006), the dissolution of potassium‐containing minerals increased after the inoculation with *Bacillus*, resulting in an improvement in the plant accessibility of soil potassium. Moreover, soil nitrogen fixed by *Bacillus* has also been reported (Lucas García, Probanza, Ramos, Colón Flores, & Gutiérrez, 2004). Although the combination of *Bacillus* in this study affected soil nutrient indicators, it did not change soil pH (Table 2). This feature is conducive to the application of the bacteria consortium in organic blueberry cultivation with special requirements on soil pH (Harmer, 1945). Few studies have shown the effects of *Bacillus* on plants under acidic conditions. The results of this study suggest that both the single *B. amyloliquefaciens* JC65 and the *Bacillus* consortiums in this study are able to function in acidic soils and deliver beneficial effects to plants and soil.

Another important indicator of soil fertility is the organic matter content. Haynes increased the yield of blueberries by applying soil conditioners such as peat and pine bark, which significantly increase soil organic matter content (Haynes & Swift, 1986). Farooque, Zaman, Schumann, Madani, and Percival (2012) also showed that, when planted in soil with significant spatial variability, blueberry yield positively correlated to soil organic matter of the specific site. Our results showed that addition of *Bacillus* positively contributes to the increase of soil organic matter (Figure 3). The increased soil organic matter content in the beneficial microbe motivated organic planting systems (S/M1/M2/M3) can thus explain the increase of blueberry yield. Specifically, Wang et al. (2019) reported that *B. cereus* AR156 can induce the secretion of organic acids, including lactic acid and caproic acid, from tomato roots, thereby promoting the growth and metabolism of root‐dwelling microorganisms. It suggested an important method that bacteria employ to improve their viability in barren soil, while consequently promoting soil organic matter. Moreover, the salinization of the soil, often caused by overuse of chemical fertilizers, leads to the risk of salt damage when the soil salinity exceeds the salt tolerance threshold of the plant. The soil EC value was reduced after bacteria treatment, indicating an effective effect of microbial agent on preventing soil salinization (Table 2). PGPRs have been developed as one of the strategies to decrease the toxic effect caused by high salinity. Besides inducing plant systemic tolerance to the salt stress, PGPRs are able to promote plant growth by facilitating plant nutrient uptake (Kohler, Caravaca, Carrasco, & Roldan, 2006), which can possibly result in the decrease of soil EC value in this study.

Compared with the single bacteria treatment, the organic planting system supplemented with three‐bacteria complex JC03 + JC65 + HS10 showed higher efficiency in improving blueberry yield (Figure 1), fruit quality nutrients (Figure 2), and maintaining soil nutrients (Figure 3). The superiority of the composite microbes could be simply due to an additive effect, a synergistic activity, or both. In order to make better use of environmental resources and gain advantage in competition, microbes have differentiated into unique material decomposition systems and acted differently in disease control (Yu et al., 2017). Thus, an additive effect is theoretically rational to achieve by mixing bacteria with different functions. Furthermore, bacteria extensively interact with each other in comprehensive community such as soil environment. Quorum sensing system by a cycle of recognizing, responding to endogenous and exogenous extracellular signals as well as secreting its own signals is a universal mechanism employed by bacteria and thereby creating interactions and regulations on different cell fates and activities (Cloud‐Hansen et al., 2006). For instance, it has been proved that biofilm formation is critical for the successful colonization and biological control efficiency of *B. subtilis* (Chen et al., 2013). Biofilm formation of *B. subtilis* can be stimulated by lactose, which is governed by quorum sensing system (Duanis‐Assaf, Steinberg, Chai, & Shemesh, 2016). Additionally, microbial peptidoglycan and cell wall fragments can act as an important signaling molecule to benefit other bacteria (Cloud‐Hansen et al., 2006). *B. cereus* and its purified peptidoglycan can promote the growth of other bacterial population by being used as a carbon source for growth (Peterson, Dunn, Klimowicz, & Handelsman, 2006). Besides the universal interaction mechanism, *Bacillus* owns its unique interaction within the genus. It has been shown that, among the tested strains, most of bacteria with the ability to promote biofilm formation of *B. subtilis* through interspecies interaction are members of *Bacillus* own genus (Shank et al., 2011). Together, the composite microbial agents in the M1/M2/M3 treatments in this study may not only act in an additive matter; instead, the combination of *Bacillus* may show a synergistic effect through comprehensive interspecies interactions. It seems promising that the compounding of microbe is a potential means for efficient growth promoting, disease prevention, and soil improvement in organic agriculture practice.

## 5 CONCLUSIONS

Based on the results, we conclude that addition of beneficial microbe in organic blueberry production significantly promotes plant growth and improves blueberry fruit quality. Interestingly, by combination of different beneficial microorganism, the promotion effect on blueberry production was enhanced significantly, indicating synergistic activity among specific mixture of bacteria. The soil retention ability of the beneficial microbe is also shown, especially for increase of soil organic matter. The change of soil nutrients may be one of the reasons that addition of bacteria affects the growth and quality of blueberry. Further studies should be focused on screening for more effective bacterial consortium and studying the interactions among the mixed species causing this synergistic activity.

## CONFLICT OF INTEREST

All authors declare no conflict of interest.

## ETHICAL APPROVAL

No human subjects or vertebrate animals were used in this study.
